# Mitochondrial Dysfunction in Astrocytes: A Role in Parkinson’s Disease?

**DOI:** 10.3389/fcell.2020.608026

**Published:** 2021-01-07

**Authors:** Collin M. Bantle, Warren D. Hirst, Andreas Weihofen, Evgeny Shlevkov

**Affiliations:** Neurodegenerative Diseases Research Unit, Biogen, Cambridge, MA, United States

**Keywords:** mitochondria, astrocyte, Parkinson’s disease, inflammation, cGAS/STING pathway, NLRP3, PINK1/Parkin pathway

## Abstract

Mitochondrial dysfunction is a hallmark of Parkinson’s disease (PD). Astrocytes are the most abundant glial cell type in the brain and are thought to play a pivotal role in the progression of PD. Emerging evidence suggests that many astrocytic functions, including glutamate metabolism, Ca^2+^ signaling, fatty acid metabolism, antioxidant production, and inflammation are dependent on healthy mitochondria. Here, we review how mitochondrial dysfunction impacts astrocytes, highlighting translational gaps and opening new questions for therapeutic development.

## Introduction

Parkinson’s disease (PD) is the second most common neurodegenerative disease after Alzheimer’s disease (AD) and the most common movement disorder worldwide (Dorsey et al., 2018). PD affects approximately 1 million Americans alive today, and the US National Institute of Neurological Disorders and Stroke (NINDS) predicts that 50,000 new cases of Parkinson’s disease are diagnosed in the US each year (Marras et al., 2018). With the aging of the Western World, the burden of this disease is set to rise tremendously over the next decade. Clinically, PD is primarily characterized by tremor, bradykinesia, rigidity, and postural instability. The main pathological hallmarks are degeneration of dopaminergic (DA) neurons of the substantia nigra pars compacta (SNpc) and presence of Lewy bodies and neurites that consist mainly of α-synuclein (α-syn) aggregates. In addition to dysfunctional α-syn proteostasis, neuroinflammatory glial activation, mitochondrial dysfunction, and oxidative stress have also been implicated in PD pathogenesis.

PD is mostly an idiopathic disorder with an age-related increase in incidence. Historically, exposure to pesticides and viruses have been linked to increased incidence of disease; however, lack of geographic clusters with epidemiological studies goes against environmental toxins or viral infections as being the primary cause of sporadic PD (Pang et al., 2019). Identification of rare autosomal dominant and recessive forms of PD in the 1990s suggested a broad contribution of genetics to PD etiology. Recent genome-wide association studies (GWAS) have provided new genetic insights into the disease etiology, strengthening the possibility of specific gene variants playing a role in PD pathogenesis (Nalls et al., 2019). To date, there are 90 significant known independent genome-wide risk signals that explain 16–36% of PD heritability, leaving a large portion of cases unexplained (Nalls et al., 2019). Research is just beginning to elucidate coalescing molecular pathways and mechanisms among different forms of PD, and accumulating evidence suggests that PD is linked to combinatorial interactions between genetic risk factors, pathogens, exposure to environmental toxins, and aging. Thus, aging, genetics, and environmental stressors each alone are unlikely to initiate PD but together may be able to induce disease (Johnson et al., 2019).

Importantly, several lines of evidence converge on mitochondrial dysfunction as a common central pathway that could integrate the pathobiological processes of sporadic and genetic PD (Cabezas et al., 2018; De Miranda et al., 2018; Gegg and Schapira, 2018; Sliter et al., 2018; Sun et al., 2018; Pang et al., 2019; Russo et al., 2019). Toxins like paraquat and rotenone have been linked to PD and act on the mitochondrial respiratory chain. Genetic studies have shown that loss of function mutations in the gene PINK1, a mitochondrial kinase, and Parkin, a cytoplasmic E3 ubiquitin ligase, cause autosomal recessive PD. PINK1 and Parkin work together in a pathway to remove damaged mitochondria by mitophagy (Pickrell and Youle, 2015). Mitophagy is the selective degradation and clearance of defective mitochondria by autophagy following mitochondrial damage or stress (Pickles et al., 2018). Mitophagy can eliminate dysfunctional mitochondria to maintain mitochondrial homeostasis and protect against neuroinflammatory activation induced by ROS and pathogen-/damage-associated molecular patterns (PAMPs/DAMPs) (Youle, 2019). Moreover, among the 90 risk alleles identified in previous GWAS studies, many of the risk variants seem to directly and indirectly impact cellular degrading pathways and other pathways related to mitochondrial functions. Finally, aging is a major risk factor, and mitochondrial dysfunction is a hallmark of aging. Thus, one hypothesis is that excessive mitochondrial damage, as observed in PINK1 and Parkin mutants, likely contributes to the degeneration of the nigrostriatal system.

Increasing evidence suggests that astrocytes play a significant role in the progression of PD (Liddelow and Barres, 2017; Clarke et al., 2018; Bantle et al., 2019; Caggiu et al., 2019; di Domenico et al., 2019; Filippini et al., 2019; Harischandra et al., 2019). Within the central nervous system (CNS), astrocytes represent over 30% of all cells and are the most abundant cell type in the brain. While early descriptions of these cells labeled them as the “glue of the brain” with a primarily passive structural role, contemporary research is shedding light to many more functions of glia in the developing and adult brain. Multiple risk alleles that have been identified in the most recent GWAS studies and previous candidate gene studies show cellular penetrance in astrocytes (Booth et al., 2017). Astrocytes function to support neuronal homeostasis, participate in the maintenance of the blood–brain barrier (BBB), and are dynamic regulators of the neuronal synaptic communication and cerebral blood flow. They also provide continuous trophic support and energy metabolism to neurons by secreting glial-derived neurotrophic factor (GDNF), regulating extracellular ion balance in the CNS, and shuttling lactate and glutamine to neurons (Sofroniew and Vinters, 2010). Additionally, although microglia have been previously thought of as the primary inflammatory cell in the CNS, inflammatory activation of astrocytes is often more persistent than microglia and is believed to be important in chronic inflammatory activation associated with PD (Saijo et al., 2009).

Recent research indicates that mitochondria regulate essential astrocyte functions, including glutamate regulation, Ca^2+^ signaling, fatty acid metabolism, transmitophagy, antioxidant production, and neuroinflammatory activation (De Miranda et al., 2018; Mouton-Liger et al., 2018; Sliter et al., 2018; Ho et al., 2019; Pang et al., 2019; Russo et al., 2019). Therefore, one hypothesis is that mitochondrial dysfunction in astrocytes impacts dopaminergic neuronal health through a gain of inflammatory function but also via loss of supportive functions, including trophic and antioxidant support. Preserving astrocytic mitochondrial function therefore may represent a disease-modifying a to slow the progression of PD. In this review, we discuss precisely how mitochondrial dysfunction in astrocytes may contribute to Parkinson’s disease and suggest new avenues for therapeutic development.

## Astrocyte Mitochondrial Function and Dysfunction in PD

Mitochondria are highly dynamic organelles with a plethora of functions, including regulation of calcium homeostasis, energy metabolism, and inflammatory activation (Winklhofer and Haass, 2010). While the majority of research investigating mitochondrial dysfunction in PD has been focused on neurons, recent studies suggest that mitochondrial dysfunction in astrocytes likely play a role in PD as well. Key astrocyte functions such as glutamate–glutamine cycle, regulation of Ca^2+^ metabolism, fatty acid metabolism, and regulation of innate immunity are dependent on functional mitochondria. Genes implicated in autosomal-recessive PD such as DJ-1 are highly expressed in astrocytes. Moreover, astrocytes can play a role in mitochondrial quality control of striatal axons via transmitophagy. While the field is still emerging, collective evidence presented here suggests that dysfunctional mitochondria in astrocytes can play a pivotal role in the progression of PD. The following sections of this review highlight the current literature supporting mitochondrial dysfunction in astrocytes as a contributing factor in PD pathophysiology.

### Astrocyte Mitochondria and Glutamate Metabolism

Magnetic resonance spectroscopy has shown disbalances in GABA-ergicas well as glutamatergic signaling in the thalamus, pons, basal ganglia, substantia nigra, and cortical regions in PD patients. Cortical glutamatergic and substantia nigra dopaminergic afferents converge onto the dendrites of medium spiny neurons in the striatum/caudate putamen where they act to modulate motor and cognitive functions (Mahmoud et al., 2019), and many so-called “axial” motor symptoms appear as a consequence of dysregulations in GABA/glutamatergic neurotransmitter systems (O’Gorman Tuura et al., 2018). One of the fundamental tasks of all astrocytes, including those in the striatum, is glutamate reuptake via glutamate uptake transporters, such as excitatory amino acid transporter (EAAT). Exacerbated activation of glutamate receptors can lead to excitotoxicity, and the balance between physiological and toxic levels of glutamate are largely controlled by astrocytes at the level of the synaptic cleft (Armada-Moreira et al., 2020). After synaptic release of glutamate, it is estimated that only 20% is absorbed by postsynaptic neurons, while the majority diffuses out of the synaptic cleft for uptake by EAAT-1 and EAAT-2 transporters in astrocytes. Upon entry in the astrocyte, glutamate is metabolized into α-ketoglutarate (α-KG) and glutamine (Schousboe et al., 2013). The glutamate dehydrogenase enzyme [alpha-ketoglutarate-dehydrogenase complex (KGDHC)], which resides in the mitochondria, catalyzes the conversion of glutamate into α-KG (Figure 1.1a). KGDHC is inhibited by the mitochondrial toxin MPP+, known to induce Parkinson’s-like phenotypes and also acts a major source of reactive oxygen species (Starkov et al., 2004). The levels of KGDHC, but not much the levels of complex II or IV of the mitochondrial respiratory chain, have been reported to be decreased in basal ganglia of PD patients (Mizuno et al., 1994), raising the possibility that the metabolism of glutamate via KGDHC plays a role in the progression of the disease.

**FIGURE 1:**
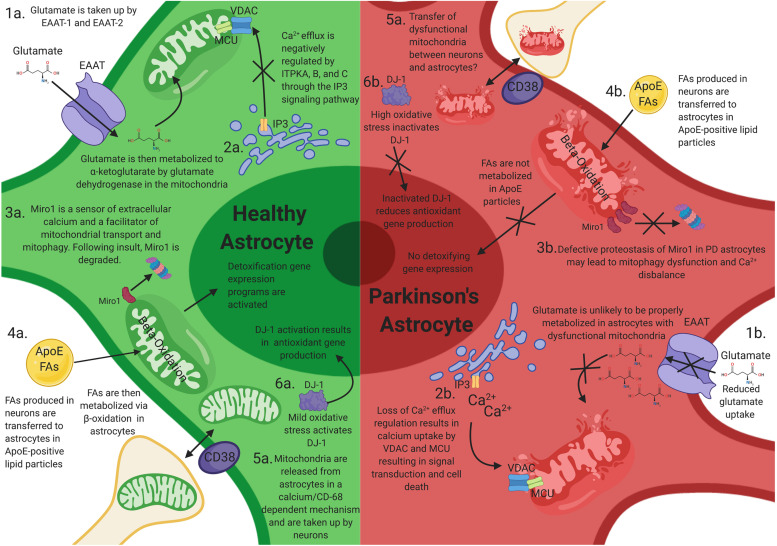
How mitochondrial dysfunction in astrocytes can contribute to Parkinson’s disease (PD) progression. Mitochondrial dysfunction in astrocytes may elicit neuronal toxicity through multiple mechanisms. **(1a)** Functional astrocyte mitochondria are needed for glutamate regulation and metabolism. **(1b)** Dysfunctional astrocyte mitochondria likely have reduced glutamate uptake and metabolism, resulting in excitatory neurotoxicity in neurons. **(2a)** Astrocytes house large stores of intracellular calcium and other ions, which is negatively regulated by ITPKA, B, and C. **(2b)** Perturbations of these calcium reservoirs results in an increase in mitochondrial intracellular calcium through VDAC1 and MCU, resulting in signal transduction and astrocytic cell death. **(3a)** Miro1 is a sensor of extracellular calcium and a facilitator of mitochondrial transport and mitophagy. Following mitochondrial stress, Miro1 is degraded, which facilitates the clearance of damaged mitochondria. **(3b)** Defective proteostasis of Miro1 in PD astrocytes may lead to mitophagy dysfunction and Ca^2+^ disbalance. **(4a)** Toxic fatty acids (FAs) are produced in neurons and are transferred to astrocytic lipid droplets by ApoE-positive lipid particles. Astrocytes then consume the FAs stored in lipid droplets via mitochondrial β-oxidation in response to neuronal activity and turn on a detoxification gene expression program. **(4b)** Loss of mitochondrial function in astrocytes prevents proper metabolism of these FAs and results in FA induced toxicity **(4b)**. **(5a,b)** Astrocytes serve as the primary cell type responsible for the clearance and transfer of damaged and healthy mitochondria to and from neurons. The transfer is completed in a calcium-dependent manner, regulated by CD38. During transmitophagy, mitophagy is thought to begin in neurons and be completed in astrocytes. **(6a)** Mild mtROS induces DJ-1 activation, which results in antioxidant gene transcription. **(6b)** High levels of mtROS inactives DJ-1 and prevents antioxidant gene transcription.

Glutamate can also be converted by astrocytes into glutamine via the glutamine synthetase enzyme. Glutamine is transported to presynaptic terminals via glutamine transporters to be converted back to glutamate by the mitochondrial enzyme glutaminase (Ortinski et al., 2010; Schousboe et al., 2013). The dysregulation of glutamine cycle is caused by reactive astrocytosis. Experimentally induced astrocytosis, by high-titer AAV2/5-GFP transduction, induced deficits in inhibitory signaling in the mouse hippocampus and enhanced excitability as a consequence to the downregulation of astrocytic glutamine synthetase (Ortinski et al., 2010). Importantly, mitochondrial dysfunction in astrocytes induces alterations in glutamate metabolism and excitotoxicity. In an elegant study, Murru and colleagues recently showed that an astrocyte-specific deletion of mAAA protease, an enzyme involved in mitochondrial quality control and proteostasis, resulted in aberrant astrocyte morphology, altered expression of EAAT-2, and a reactive inflammatory signature (Murru et al., 2019; Figure 1.1b), pointing to a common mechanism. A recent study has suggested an additional link of glutamate dyshomeostasis to PD (Vallerga et al., 2020), where hypermethylation in PD is associated with downregulation of the SLC7A11 gene. SLC7A11 codes for a cysteine-glutamate antiporter, which is predominantly expressed by astrocytes in the brain, and regulates levels of the antioxidant glutathione. This study focused on genome-wide blood-based DNA methylation data, and further experiments are required to determine if there is similar epigenetic control in the brain. However, reduced levels of glutathione have been reported in the substantia nigra in PD patients compared with aged-matched controls (Sian et al., 1994), which could be attributed to a pathological downregulation of the cysteine–glutamate antiporter, potentially via this mechanism. Therefore, alterations in the levels of glutamate and its metabolic intermediaries, as seen in PD, can be one of the pathological outcomes of astrocytic mitochondrial dysfunction.

### Astrocyte Mitochondria and Intracellular Ca^2+^ Regulation

One of the key features of mitochondria is their ability to regulate cellular Ca^2+^ concentrations (Bagur and Hajnoczky, 2017). A major source of mitochondrial Ca^2+^ is the endoplasmic reticulum (ER), where Ca^2+^ efflux is regulated by canonical G-coupled receptor/inositol triphosphate (IP3) signaling pathway. The IP3-gated Ca^2+^ efflux in astrocytes can be negatively regulated by the inositol triphosphate kinase ITPKB—a kinase that phosphorylates the 3′ position of inositol-1,4,5-triphosphate (IP_3_) to generate inositol 1,3,4,5 tetrakisphosphate (IP_4_) (Communi et al., 1999; Miller et al., 2015). The mitochondrial transmembrane protein voltage-dependent anion-selective channel (VDAC) mediates the transfer of Ca^2+^ to the mitochondrial intermembrane space. Once Ca^2+^ enters the mitochondria, the ion is channeled to the matrix via the mitochondrial calcium uniporter (MCU) complex. MCU resides in the mitochondrial inner membrane and consists of the MCU transmembrane channel and two regulatory subunits, MICU1 and MICU2. A brain/specific isoform (MICU3) has also been described (Kamer et al., 2018). Notably, recent GWAS studies have identified a signal in the ITPKB locus as well as the MICU3 locus as risk factors for Parkinson’s disease (Chang et al., 2017; Nalls et al., 2019). Given that both ITPKB and MICU3 are expressed in astrocytes, it is plausible that dysregulated mitochondrial calcium uptake in astrocytes can play a role in the progression of PD (Figure 1.2). The precise contribution of intracellular Ca^2+^ stores to overall Ca^2+^ signaling in astrocytes, especially in astrocyte processes, is nevertheless controversial. Ca^2+^-dependent release of gliotransmitters have been implicated in learning and memory, but knockout of IP3R2, the main isoform found in astrocytes, produced no changes in anxiety or motor behavior, and no changes in learning and memory were observed (Petravicz et al., 2014). Extracellular Ca^2+^ in the astrocyte processes can arrive via transient receptor potential A1 channels (Shigetomi et al., 2011), reverse sodium-calcium exchange (Gerkau et al., 2018), and perhaps N-methyl-D-aspartate receptors (NMDARs) (Stephen et al., 2015).

Miro1/RhoT1 is a component of the mitochondrial motor/adaptor complex with Ca^2+^ EF sensing hands and has been shown to participate in the localization of mitochondria close to sources of extracellular Ca^2+^ (Macaskill et al., 2009; Wang and Schwarz, 2009). In an elegant study, Stephen et al. showed that Miro1 also localizes mitochondria adjacent to sources of extracellular Ca^2+^ in astrocytic processes (Stephen et al., 2015). Interestingly, Miro1/RhoT1 is a target of the PINK1/Parkin pathway for mitochondrial quality control (Wang et al., 2011; Shlevkov et al., 2016), and defective proteostasis of Miro1 has been observed in human-induced pluripotent stem cell (hIPSC) lines derived from PD patients (Hsieh et al., 2019). It is possible that defective Miro1 turnover in PD astrocytes can lead to disbalanced Ca^2+^ signaling in astrocytic processes (Figure 1.3).

Astrocytes *in vivo* typically show cellular structures called lamellar sheets as well as peripheral astrocytic processes, both of which contain mitochondria (Jackson and Robinson, 2018). Spontaneous, cell-autonomous Ca^2+^ spikes have been recently observed in these microdomains (Khakh, 2019). It is plausible that Ca^2+^ transients can regulate glucose mobilization (Howarth, 2014) and influence the activity of neighboring neurons and glia by the release of ATP, D-serine, and glutamate (Haydon, 2001). Astrocyte Ca^2+^ transients have been shown to occur more frequently following CNS injury (Kuchibhotla et al., 2009). Importantly, mitochondria are the key mediator of spontaneous Ca^2+^ increases in astrocytes *in vivo* independently of Ca^2+^ release from ER stores in a mechanism that involves the transient opening of the permeability transition pore (mPTP) (Agarwal et al., 2017). Mitochondrial Ca^2+^ uptake in astrocytes is not only involved in homeostatic regulation of astrocytic functions but also plays a role in the astrocyte response to acute injury. Gbel at el. recently found that acute injury and blood–brain barrier disruption trigger the formation of a prominent mitochondria-enriched compartment in astrocytic endfeet, enabling vascular remodeling (Gbel et al., 2020). Vascular remodeling in this model was dependent on mitofusin 2 and mitochondria–ER contact sites. These structural changes were mirrored by impaired mitochondrial Ca^2+^ uptake leading to abnormal cytosolic transients within endfeet. Since mitochondrial remodeling happens as a general response to injury in astrocytes (Motori et al., 2013), the precise cellular mechanisms of astrocyte reaction to injury via mitochondria may also be relevant for PD. Finally, Ca^2+^ transients in astrocytic processes have also been implicated directly in synaptic transmission (Di Castro et al., 2011; Panatier et al., 2011); however, whether mitochondrial Ca^2+^ uptake can directly regulate synaptic transmission has yet to be established. Taken together, these studies suggest that mitochondrial dysfunction in astrocytes can lead to perturbations of Ca^2+^-mediated astrocyte functions as well as reinforce a maladaptive response to injury. Emerging evidence suggests that specific alterations of ITPKB, MICU3 and Miro1 functions are predicted to result in altered mitochondrial Ca^2+^ handling in astrocytes and thereby contribute to PD progression.

### Astrocyte Mitochondria and Fatty Acid Metabolism

Mitochondria also serve as a metabolic hub in the cells, and metabolic coordination between neurons and astrocytes is critical for the health of the brain. Recent research from Jie Liu’s laboratory has shown that toxic fatty acids (FAs) produced in hyperactive neurons are transferred to astrocytic lipid droplets by ApoE-positive lipid particles (Ioannou et al., 2019). Astrocytes then consume the FAs stored in lipid droplets via mitochondrial β-oxidation in response to neuronal activity and turn on a detoxification gene expression program (Figure 1.3a). Therefore, FA metabolism is coupled in neurons and astrocytes to protect neurons from FA toxicity during periods of enhanced activity. Mitochondrial dysfunction in astrocytes likely decreases FAs metabolism (Figure 1.3b). Interestingly, fatty acids, as well as lipid droplets, have been associated to α-syn toxicity in cellular and animal models of PD (Vincent et al., 2018; Fanning et al., 2019). Using genetic screens for suppressors of α-syn toxicity in yeast, two laboratories independently identified stereoacyl-CoA-desaturase 1 (SCD1) as a mediator of α-syn toxicity. α-syn elevation increased the levels of oleic acid, which accumulated in lipid droplets in yeast and in neurons. SCD1 mediates the conversion of stearic acid to oleic acid—and its inhibition reduced the levels of oleic acid and, concomitantly, the toxicity of α-syn in a variety of models. Given that break down, via beta-oxidation, of oleic acid happens in astrocytic mitochondria and beta-oxidation in astrocytes is activated in model systems with elevated fatty acids (Ioannou et al., 2019), it is plausible that astrocytic mitochondria play a key role in mitigating the toxicity of α-syn in PD brains.

### Astrocyte Mitochondria Transfer and Transmitophagy

Advances in imaging techniques have enabled demonstration of mitochondrial transfer between neurons and astrocytes in the context of injury. The phenomenon of transcellular mitophagy (transmitophagy) was first demonstrated in the mouse optic nerve tract (Davis et al., 2014), where basal mitophagy of axonal mitochondria was shown to occur primarily in neighboring astrocytes. Similar processes are likely to happen elsewhere in the brain (Davis et al., 2014). A recent paper has presented elegant evidence suggesting that transneuronal mitophagy occurs *in vivo* in PD models (Morales et al., 2020). Here, astrocytes serve as the primary cell type responsible for the clearance of damaged mitochondria—a concept highly relevant in the context of PD associated to Parkin and PINK1 loss of function mutations. Notably, PINK1 activity was recently predominately found in astrocytes while almost absent in neurons (Barodia et al., 2019). The phenomenon of transcellular mitophagy may also point at novel therapeutic avenues. For example, enhancing PINK1/Parkin-mediated mitophagy specifically in striatal astrocytes may help alleviate the burden of damaged mitochondria in dopaminergic neurons.

Moreover, the converse has also been observed: astrocytes can transfer healthy mitochondria to axons in the context of injury (Hayakawa et al., 2016; Joshi et al., 2019; Figures 1.4a,b). In this model, astrocytes release mitochondria in a calcium-dependent mechanism involving CD38 and cyclic ADP ribose signaling and are then taken up by neurons. Recently, Cheng et al. used human-induced pluripotent stem cells to show that iPSC-derived astrocytes can act as donors of mitochondria and rescue dopaminergic neuronal toxicity in coculture systems (Cheng et al., 2020). Others have also demonstrated how astrocytic mitochondria can alleviate neuronal toxicity. For example, coculture of cisplatin-treated neurons with astrocytes increased neuronal survival, restored neuronal mitochondrial membrane potential, and normalized neuronal calcium dynamics especially in neurons that had received mitochondria from astrocytes, which underlines the importance of mitochondrial transfer (English et al., 2020). These beneficial effects of astrocytes were associated with transfer of mitochondria from astrocytes to cisplatin-treated neurons. In this model, small interfering RNA (siRNA)-mediated knockdown of the Rho-GTPase Miro-1 in astrocytes reduced mitochondrial transfer from astrocytes to neurons and prevented the normalization of neuronal calcium dynamics (Fu et al., 2020). Whether similar processes occur *in vivo* in the striatum/caudate putamen is an exciting avenue of research and can point to novel therapeutic interventions.

### Astrocyte Mitochondria, Reactive Oxygen Species, and DJ-1

Oxidative stress is an important pathogenic factor in PD. Despite neurons being highly dependent on oxidative metabolism, they display limited defense mechanisms against oxidative stress compared to astrocytes. Astrocytes play a key role in controlling redox homeostasis in the brain (Fernandez-Fernandez et al., 2012), and the adaptive response of astrocytes to oxidative stress seems indispensable to maintain redox homeostasis in the brain (Liddell, 2017).

Mitochondria are a major source of reactive oxygen species (ROS) in the cell as a byproduct of the electron transport chain activity. Damaged mitochondria-induced oxidative stress is a well-known contributor to neurodegeneration. ROS occur mainly at complexes I and III of the respiratory chain, and ROS production increases when the electron transport chain is compromised, leading to a leakage of electrons, which react with oxygen to form superoxide. ROS can change mitochondrial metabolism (Nemoto et al., 2000), and the production of excess superoxide can cause oxidative DNA damage and genomic instability (Samper et al., 2003). Mitochondrial DNA (mtDNA) is highly susceptible to damage because it is very close to the source of ROS, is not protected by histones, and DNA repair capacity in mitochondria is low. Mutations in mtDNA can, in turn, make mitochondria produce more ROS, initiating a self-perpetuating vicious cycle (Hahn and Zuryn, 2019). Importantly, mutations in mtDNA have been observed in PD patients (Simon et al., 2004; Lin et al., 2012), although they seem to preferentially accumulate in neurons (Cantuti-Castelvetri et al., 2005).

Furthermore, DJ-1 point mutations and gene deletions are one of the causes of autosomal-recessive PD (PARK7) (Bonifati et al., 2003; Honbou et al., 2003; Ariga et al., 2013). In brain tissue obtained from sporadic PD patients, DJ-1 is strongly upregulated in reactive astrocytes but not in neurons (Bandopadhyay et al., 2004). DJ-1 protects against metal-induced neurotoxicity and regulates intracellular antioxidant stress responses through the transcription factor Nrf2 (nuclear factor-like 2) (Dolgacheva et al., 2019), and mitochondrial localized DJ-1 is thought to be cytoprotectant against oxidative-stress-induced cell death (Bjorkblom et al., 2013; Figure 1.6).

During oxidative stress, DJ-1 can be oxidized at position 106 (Cys106) (Repici and Giorgini, 2019). DJ-1 oxidation has been reported in patients with Parkinson’s disease (PD), and oxidized DJ-1 is consistently observed in astrocytes (Repici and Giorgini, 2019). Although the relationship between DJ-1 oxidation and PD is still unclear, some have attempted to use astrocytic oxidized DJ-1 as a biomarker of PD (Repici and Giorgini, 2019). In mice, DJ-1 deficiency induces enhanced sensitivity of dopaminergic neurons to oxidative stress, and DJ-1 KO mice also suffer deficient glutamate uptake, which can induce excitoneurotoxicity and neurodegeneration (Kim et al., 2005, 2013, 2016; Booth et al., 2017). A more recent study found that deficiency in DJ-1 in mice delays neuronal repair due to a decrease in the astrocytes specific chemokine CCL2/MCP-1 (Choi et al., 2020), and that astrocytic DJ-1 may regulate inflammatory activation in astrocytes (Waak et al., 2009). Together, these studies indicate that mitochondrial ROS impacts DJ-1 function in astrocytes, which in turn can contribute to PD progression.

## Role of Astrocytic Mitochondria as Inflammatory Mediators in PD

Inflammatory activation of astrocytes contributes to the neuropathology induced by mitochondrial toxins rotenone, paraquat, and 1-methyl-4-phenyl-1,2,3,6-tetrahydropyridine (MPTP) (Cabezas et al., 2018; De Miranda et al., 2018; Kirkley et al., 2019). ROS, mitochondrial DNA (mtDNA), and ATP, all produced primarily by mitochondria, are noxious and inflammatory stimuli to astrocytes. Recent evidence indicates that mitochondria are at the center of innate immunity pathways with relevance to neurodegeneration such as NLR family, pyrin domain containing 3 (NLRP3)-inflammasome pathway and cyclic guanosine monophosphate–adenosine monophosphate cGAMP synthase (cGAS)/stimulator of interferon gene (STING) pathway. In this section, we highlight the evidence supporting the notion that damaged mitochondria are a major source of neuroinflammatory signals.

### Mitochondrial Dysfunction and NLRP3 Activation

The NLRP3 inflammasome is a pattern recognition receptor activated in response to a variety of pathogen-derived and endogenous stimuli. Upon activation, NLRP3 forms a heptameric ring that binds ASC and procaspase 1, cleaving and activating caspase-1, which results in the maturation and secretion of the proinflammatory cytokines interleukin (IL)-1β and IL-18. The NLRP3 inflammasome is present in microglia and astrocytes in the CNS (Song et al., 2017). Mitochondria are closely connected to the activation of the inflammasome. Mitochondrial disruption caused by NLRP3 stimuli leads to the generation of mitochondrial ROS and release of mtDNA to the cytoplasm (Nakahira et al., 2011; Zhou et al., 2020). Oxidized mitochondrial DNA can reinforce NLRP3 activation and enhance IL-1β secretion (Shimada et al., 2012). Zhou et al. have shown that, upon a Toll-like receptor 4 (TLR4) lipopolysaccharide (LPS) stimulation, nuclear factor kappa B (NF-κB) induces the expression of NLRP3 and pro-IL-1β during inflammasome priming, which activates interferon regulatory factor 1 (IRF1) and induces the expression of the nucleoside monophosphate kinase cytidine/uridine monophosphate kinase 2 (CMPK2) (Zhou et al., 2020). CMPK2 then locates to the outer mitochondrial membrane and increases mtDNA synthesis. Oxidized mtDNA can interact with the NLRP3 to induce IL-β. Subsequent escape of mtDNA from the cell is a potent extracellular inflammatory stimulus to astrocytes. Upon mtDNA binding to TLR9 on astrocytes, NF-κB translocates to the nucleus and drives astrocytic-specific inflammatory cytokine and chemokine transcription of CCL2, Cxcl10, IL-6, and IL-1β (Choi et al., 2014).

PINK1/Parkin-mediated mitophagy is a key mechanism to mitigate mitochondrial damage, and incomplete mitophagy can trigger NLRP3 and other inflammatory pathways (Figures 2.2a,b; Gkikas et al., 2018). Deficiency in LC3B-, ATG5-, ATG16L1, and Beclin in macrophages results in increased levels of cytosolic levels of mtDNA and mtROS, which triggers NLRP3-dependent IL1-β secretion (Saitoh et al., 2008; van Bruggen et al., 2010). NLRP3 can also be activated by the mitochondria-specific lipid cardiolipin. Mitochondrial depolarization translocates cardiolipin from the inner mitochondrial membrane (IMM) to the outer mitochondrial membrane (OMM) where it associates to NLRP3 (Iyer et al., 2013). An attractive hypothesis is that, in PINK1 and Parkin mutant astrocytes, elevated levels of mtROS and cardiolipin result in sustained activity of the NLRP3 inflammasome (Figure 2.1b). Notably, there are also reports indicating that, *in vitro*, PINK1/Parkin mitophagy can be inhibited upon inflammasome activation, since Parkin can be cleaved by caspase-1, and caspase-8, possibly to facilitate maximal activation of the mitochondria-associated NLRP3 activity (Kahns et al., 2003; Yu et al., 2014). Collectively, these studies demonstrate the crucial role that mitochondria play in the activation of NLRP3 and underline PINK1/Parkin pathway of mitophagy as a key mechanism limiting excessive inflammation and preserving CNS homeostasis.

**FIGURE 2:**
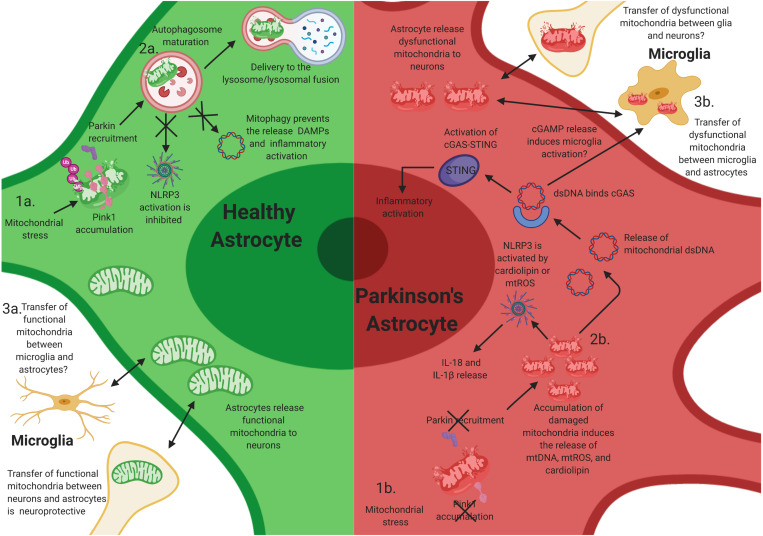
Mitochondrial dysfunction in astrocytes contributes to neuroinflammatory activation of astrocytes. Mutations in PINK1 and Parkin likely elicit neuroinflammatory activation in astrocytes. **(1a)** After mitochondrial stress, PINK1 protein accumulates on the outer mitochondrial membrane. Phosphorylation of polyubiquitin and Parkin induces autophagosome maturation and eventually delivery to the lysosome. **(1b)** Loss-of-function (LOF) mutations in PINK1 and Parkin prevent PINK1 accumulation on the mitochondrial membrane and the activation of Parkin during mitochondrial stress, resulting in the accumulation of damaged mitochondria within astrocytes. This likely facilitates chronic inflammatory activation through multiple inflammatory pathways, including NLRP3 activation. **(2a)** Functional mitophagy prevents the release of damage-associated molecular patterns (DAMPs)/pathogen-associated molecular patterns (PAMPs) and activation of the NLR family, pyrin domain containing 3 (NLRP3) inflammasome in astrocytes. **(2b)** The accumulation of damaged mitochondria likely results in the release of intracellular DAMPs/PAMPs, cardiolipin, and mtROS, which activates NLRP3 and the cyclic guanosine monophosphate–adenosine monophosphate cGAMP synthase (cGAS)/stimulator of interferon genes (STING) pathway, resulting in inflammatory activation and release of interleukin (IL)-1β and IL-18, and potentially inducing the activation of microglia via extracellular release of cGAMP. **(3a)** Transfer of functional mitochondria from astrocytes to neurons is thought to be neuroprotective; however, whether microglia release functional mitochondria to astrocytes is elusive. **(3b)** Joshi et al. have shown that activated microglia can release dysfunctional and fragmented mitochondria to astrocytes. Astrocytes then transfer dysfunctional mitochondria to neurons, resulting in neuronal death. However, whether this occurs *in vivo* is unknown.

### Mitochondrial Damage and cGAS/STING Pathway

Cytosolic double-stranded DNA (dsDNA) is recognized by the cGAS/STING pathway DNA sensing system (Ablasser and Chen, 2019). Binding of double-stranded polynucleotide to cGAS activates STING to induce interferon-β (IFNβ). The cGAS/STING pathway is not expressed in neurons but is highly expressed in astrocytes and in microglia. Probably as an evolutionary consequence of its role in the detection of viral dsDNA, cGAS also recognizes mtDNA. When the mtDNA nucleoid is disrupted, mtDNA is released to the cytosol where it activates cGAS and STING-induced pathway of inflammation (Figures 2.2a,b; West et al., 2015). In addition, proapoptotic Bcl-2 family members Bax and Bak can permeabilize the outer membrane, releasing cytosolic mtDNA. Low levels of Bax and Bak activity without caspase activation can induce cytokine expression (Brokatzky et al., 2019), but upon stronger stimulus, apoptotic caspases eliminate cGAS signaling (McArthur et al., 2018; Riley et al., 2018).

Recent data from the Youle lab has shown that the PINK1/Parkin pathway of mitophagy also mitigates unwanted cGAS/STING activation (Sliter et al., 2018; Figure 2.1). Mice that lack either PINK1 or Parkin do not display parkinsonism-related phenotypes. However, if Parkin KO mice are crossed to mitochondrial DNA mutator mice (a mouse model of stress resulting from mtDNA mutation accumulation), these mice show loss of nigrostriatal neurons as well as L-DOPA rescuable motor deficits (Pickrell et al., 2015). Notably, these mice have increased serum levels of mtDNA as well as IL-6 and IFNβ signaling mediated by the cGAS/STING pathway (Sliter et al., 2018). Moreover, preventing inflammation in this model by removal of STING prevented neurodegeneration. However, a caveat is that it has not yet been shown if mtDNA release is controlled by mitophagy or if it is a mitophagy-independent function of PINK1/Parkin pathway. Further, it is still unclear which cells in this model release mtDNA and promote cGAS activation and which cells express STING and secrete cytokines. One possibility is that dysfunctional mitochondria in neurons and in astrocytes can act as a source of cGAMP, which can travel between cells (Ablasser and Chen, 2019) and activate STING in other astrocytes as well as in microglia. In this context, the recent identification of cGAMP receptor SLC19A1 is posed to advance the mechanistic understanding of neuroinflammation induced by mitochondrial damage (Luteijn et al., 2019; Ritchie et al., 2019).

### Crosstalk in Astrocyte Inflammatory Activation

These previous studies highlight the immunological roles of astrocytes in PD and hint at the importance of glial–glial and glial–neuronal crosstalk. Similar to microglia, astrocytes can be transformed into A1 neurotoxic astrocytes after activation by IL-1α, TNF, and complement component 1q (C1q) from microglia (Liddelow and Barres, 2017; Liddelow et al., 2017). Joshi et al. recently demonstrated that fragmented and dysfunctional mitochondria released from microglia can also induce A1 neurotoxic astrocytes and subsequent neuronal loss (Figure 2.3b; Joshi et al., 2019). In addition, they show that activated astrocytes also secrete dysfunctional mitochondria to neurons and that transferring conditioned media from the astrocyte cultures to primary neurons induced neuronal damage. Importantly, filtering out the mitochondria from the astrocyte conditioned media reduced neuronal death (Joshi et al., 2019). Thus, the propagation of the inflammatory response from microglia to astrocytes may be, in part, mediated by mitochondria fragments. Whether microglia have the capacity to release functional mitochondria to astrocytes, and if this mechanism translates to *in vivo* models is still elusive (Figure 2.3a).

This study, together with other work, also highlights the possible existence of a positive feedback loop between mitochondria perturbations and astrocyte inflammatory activation. For example, incomplete mitophagy from PINK1 or Parkin mutations in neurons and glia may promote mitochondrial fragmentation, enhance inflammatory responses, and mtROS in astrocytes, which then further potentiates inflammatory activation and dysfunction of neighboring cells. Considering the minimal turnover of astrocytes coupled with their long and persistent inflammatory activation, repairing dysfunctional mitochondria or removing damaged mitochondria from astrocytes may be broadly neuroprotective and beneficial against the undesired chronic neuroinflammation in PD (Gkikas et al., 2018).

## Astrocytes and Therapeutic Considerations in PD

**FIGURE 3:**
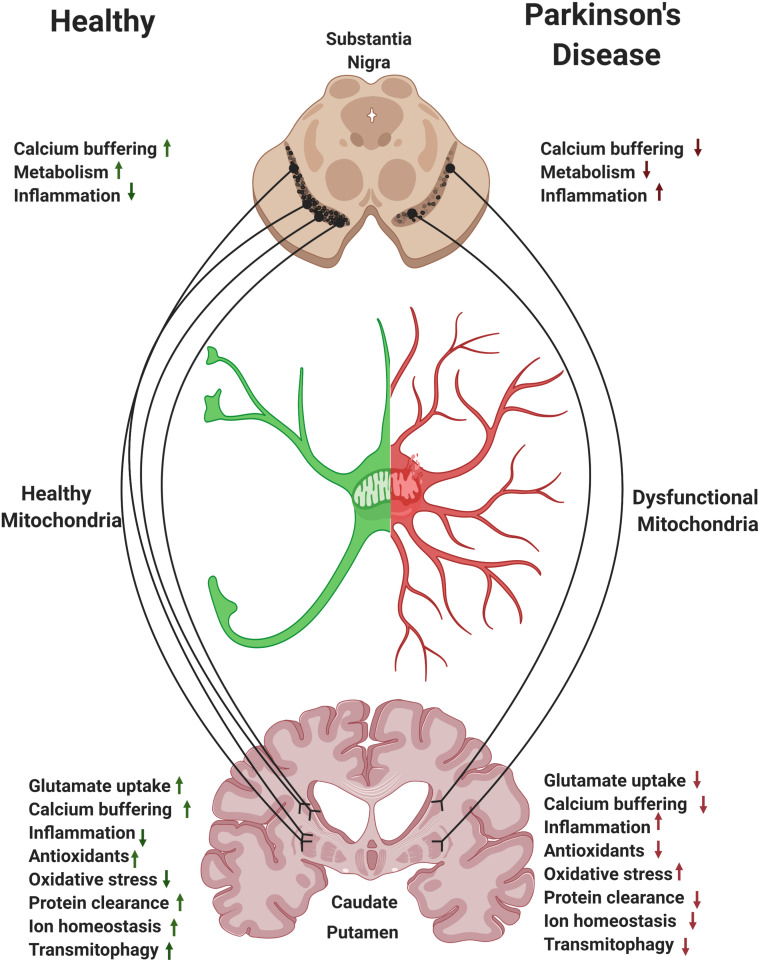
Astrocytes and the nigrostriatal pathway. Within the nigrostriatal pathway, astrocyte numbers and functions are most abundant in the striatum/caudate putamen, where dopaminergic neurons support is the highest and where loss of astrocytic function is likely to have the most detrimental effect in Parkinson’s disease (PD). Reversing mitochondrial dysfunction in astrocytes in the striatum may induce significant therapeutic benefit in PD.

Striatal astrocytes play a key role in supporting the extensive branches of the SNpc axons. Mitochondria in striatal astrocytes regulate local levels of ions, glutamate, and fatty acids and regulate inflammatory signals. Moreover, astrocytes facilitate mitophagy of axonal damaged mitochondria via transmitophagy (Figure 3; Morales et al., 2020). We hypothesize that, perhaps due to the extensive branching of SNpc axons, it is the caudate and putamen regions of the striatum where the need for astrocyte support can be the highest and where loss of astrocytic function may have the most detrimental effect in PD. Thus, modulation of mitochondrial function in striatal astrocytes may represent a disease modifying strategy for PD.

It is becoming clear that astrocytes represent a diverse population of cells with brain-area and disease-specific functions (Khakh, 2019). New techniques such as single nucleus RNA (snRNA) seq are revealing cellular diversity in many brain areas, including the basal ganglia and the striatum (Saunders et al., 2018; Zeisel et al., 2018). There are likely several distinct populations of astrocytes within the nigrostriatal pathway, and further research is required to better understand their respective physiologies, including any differential reactivity (Gokce et al., 2016). Batiuk et al. have recently identified regional specific astrocyte markers in the mouse hippocampus and cortex with variable functions and phenotypes (Batiuk et al., 2020), supporting concepts that were proposed 30 years ago (Wilkin et al., 1990). Although regional specific midbrain astrocytes have not yet been identified, studies like this one highlight the possibility of region-specific astrocyte phenotypes in PD-relevant brain regions. Future studies using single-cell and single-nuclear RNA sequencing of different neuroanatomical brain regions will likely address this possibility. Additionally, considering the cellular penetrance of PD-related gene variants in astrocytes, whether the cellular penetrance of PD-related gene mutations in astrocytes are regionally specific could readily be elucidated with single-cellular omics studies. This represents a knowledge gap and cellular “omics” studies in diseased compared to control (both young and aged) will be critical in advancing this field and increasing our understanding of the underlying pathophysiology of PD.

The rapidly evolving field of gene therapy is providing promising therapies and targeting directly astrocytes is an attractive idea. One can conceive a therapeutic approach where genes are expressed specifically in astrocytes or capsids are targeted specifically to this population. Given that astrocytes are relatively spared in PD, understanding precisely how manipulating astrocyte function represents a window of opportunity. Based on the literature reviewed above, we propose that modulating mitochondrial function in astrocytes is predicted to affect a myriad of astrocyte functions and potentially diminish their inflammatory state. Targeting these cells could promote a beneficial environment in the striatum and adjacent regions and slow the progression of the disease. With the ever-increasing amount of data supporting mitochondrial dysfunction in PD, it is reasonable to believe that preserving and replenishing mitochondrial function in astrocytes may slow the progression of PD by broadly regulating glutamate metabolism, modulating calcium signaling, regulating fatty acid metabolism, increasing transmitophagy, and reducing oxidative stress and chronic neuroinflammation. As technologies advance, astrocytes become an attractive target for precision therapeutics.

However, future studies will need to explicitly address the therapeutic potential of manipulating mitochondrial function in astrocytes. Many of the links to PD drawn in the literature are indirect, and mechanistic details are mostly obscure. Direct proof for mitochondrial dysfunction in astrocytes in human PD is incomplete, as is the involvement of this cell type in neurodegeneration in animal models. Nevertheless, we believe that further exploration of these questions are key for a comprehensive understanding of the disease and that focusing on mitochondrial dysfunction in astrocytes will pave the way for novel therapeutic avenues to disease modifying treatments.

## Author Contributions

ES conceived the manuscript and CMB created figures. All authors contributed to the article and approved the submitted version.

## Conflict of Interest

All authors are employees and shareholders of Biogen.
